# Zoonotic Transmission of *mcr-1* Colistin Resistance Gene from Small-Scale Poultry Farms, Vietnam

**DOI:** 10.3201/eid2303.161553

**Published:** 2017-03

**Authors:** Nguyen Vinh Trung, Sébastien Matamoros, Juan J. Carrique-Mas, Nguyen Huu Nghia, Nguyen Thi Nhung, Tran Thi Bich Chieu, Ho Huynh Mai, Willemien van Rooijen, James Campbell, Jaap A. Wagenaar, Anita Hardon, Nguyen Thi Nhu Mai, Thai Quoc Hieu, Guy Thwaites, Menno D. de Jong, Constance Schultsz, Ngo Thi Hoa

**Affiliations:** University of Amsterdam, Amsterdam, the Netherlands (N.V. Trung, S. Matamoros, W. van Rooijen, A. Hardon, M.D. de Jong, C. Schultsz);; Amsterdam Institute for Global Health and Development, Amsterdam (N.V. Trung, S. Matamoros, C. Schultsz);; Centre for Tropical Medicine, Ho Chi Minh City, Vietnam (N.V. Trung, J.J. Carrique-Mas, N.H. Nghia, N.T. Nhung, T.T.B. Chieu, J. Campbell, G. Thwaites, C. Schultsz, N.T. Hoa);; University of Oxford, Oxford, UK (J.J. Carrique-Mas, J. Campbell, G. Thwaites, N.T. Hoa);; Sub-Department of Animal Health, My Tho, Vietnam (H.H. Mai, T.Q. Hieu);; Utrecht University, Utrecht, the Netherlands (J.A. Wagenaar);; Central Veterinary Institute of Wageningen University & Research, Lelystad, the Netherlands (J.A. Wagenaar);; Preventive Medicine Center, My Tho (N.T.N. Mai)

**Keywords:** zoonoses, poultry farms, Vietnam, antibiotic resistance, transmission, *E. coli*, chicken, food production, colistin, fecal colonization, colistin resistance, backyard farm, small-scale farm, antimicrobial resistance, bacteria

## Abstract

We investigated the consequences of colistin use in backyard chicken farms in Vietnam by examining the prevalence of *mcr-1* in fecal samples from chickens and humans. Detection of *mcr-1*–carrying bacteria in chicken samples was associated with colistin use and detection in human samples with exposure to *mcr-1*–positive chickens.

Colistin resistance is a gradually emerging problem among gram-negative bacteria in clinical settings in many countries (*1*). A transferable plasmid-derived colistin resistance gene *mcr-1* discovered in China and subsequently found worldwide could be mediating this emergence (*2*,*3*). Use of colistin in animal production has been suggested as the most likely factor contributing to the emergence of the *mcr-1* gene (*2*). However, systematic studies applying the One Health approach to investigate the epidemiologic link between the use of colistin in agriculture and colonization with *mcr-1*–carrying bacteria in the community are lacking (*4*).

Colistin use in humans is negligible (*5*), but it is one of the most commonly used antimicrobial drugs in animal production in Vietnam (*6*). We investigated the consequences of colistin use in chicken farms by assessing chickens, farmers, and nearby persons for the presence of *mcr-1*–carrying bacteria and performing epidemiologic analyses to assess the risk for subsequent transmission to unexposed human populations in southern Vietnam.

## The Study

From March 2012 to April 2013, we conducted a systematic, cross-sectional study examining antimicrobial drug use and colonization with antimicrobial-resistant *E. coli* in chickens and humans in Tien Giang Province, Vietnam. Fecal samples from 204 chicken farms and rectal swabs from 204 chicken farmers (1 farmer/farm) were collected as described (Technical Appendix 1) (*7*,*8*). We additionally collected rectal swabs from age- and sex-matched persons not involved in poultry farming from the same districts (rural persons, n = 204) and from their provincial capitals (urban persons, n = 102) (*8*).

Samples were cultured on MacConkey plates with and without antimicrobial drugs. A sweep of the full growth on plain MacConkey plates was collected and screened for the presence of *mcr-1* by PCR as described previously (*2*). Logistic regression models were built to investigate the risk factors associated with the presence of *mcr-1* on chicken farms and in human participants. Then, we selected (using a random number table) individual *E. coli* colonies (n = 200) and extended-spectrum β-lactamase (ESBL)–producing *E. coli* colonies (n = 122) growing on different MacConkey plates and repeated PCR to confirm the presence of *mcr-1* in *E. coli* isolated from chickens and humans. We tested all *mcr-1*–positive *E. coli* isolates for colistin susceptibility using Etest (bioMérieux, Marcy l’Etoile, France) and interpreted test results in accordance with the European Committee on Antimicrobial Susceptibility Testing breakpoints (*9*). In addition, whole-genome sequencing was performed on all *mcr-1*–positive *E. coli* isolates as described (Technical Appendix 1).

From a total of 204 chicken and 510 human fecal specimens, 188 and 440 MacConkey sweeps were available for *mcr-1* screening by PCR, respectively. The adjusted prevalence of *mcr-1* was 59.4% (95% CI 47.9%–71.0%) in chicken and 20.6% (95% CI 15.9%–25.2%) in human fecal samples (Table 1).

**Table 1:** Prevalence of fecal colonization with *mcr-1*–carrying bacteria in chickens and humans, Tien Giang Province, Vietnam, 2012–2013

Source	Prevalence of fecal colonization with *mcr-1*–carrying bacteria	
	No. positive sweeps/total (%)	Adjusted prevalence, % (95% CI)
All chicken farms	93/188 (49.5)	59.4 (47.9–71.0)
Household chicken farms	53/94 (56.4)	59.5 (47.9–71.1)
Small-scale chicken farms	40/94 (42.6)	47.9 (35.4–60.3)
All human participants	84/440 (19.1)	20.6 (15.9–25.2)
All farmers	45/179 (25.1)	25.2 (18.3–32.0)
Farmers exposed to *mcr-1*–negative chickens	16/91 (17.6)	15.5 (7.7–23.3)
Farmers exposed to *mcr-1*–positive chickens	29/88 (33.0)	34.7 (23.9–45.5)
Rural persons	31/173 (17.9)	17.6 (11.6–23.7)
Urban persons	8/88 (9.1)	9.1 (3.1–15.1)

Among 200 *E. coli* isolates, *mcr-1* was detected in 10/78 (12.8%) isolates from chickens, 2/50 (4.0%) isolates from farmers, and 0/72 isolates from persons who did not farm. Similarly, *mcr-1* was detected in 9/38 (23.7%) and 1/44 (2.3%) of ESBL-producing *E. coli* isolated from chickens and farmers, respectively.

The MIC of colistin for the 22 *mcr-1*–carrying *E. coli* isolates ranged 3–4 mg/L. Because the Etest might underestimate the true MIC (*10*), these results indicate reduced susceptibility. Single-nucleotide polymorphism (SNP)–based phylogenetic analyses of the core genomes showed little genomic similarity between isolates, but the analyses did show many isolates belonged to the same multilocus sequence types (n = 14) (Figure). Analysis of the acquired resistance genes, reflecting the presence of an accessory genome, showed a large variation in resistance gene content, with only the *tet*(A) gene, encoding for tetracycline resistance, present in all genomes (Technical Appendix 2 Table). De novo bacterial genome assembly was performed, and the contigs carrying *mcr-1* were analyzed. A replication origin could be located in 5 isolates, leading to the identification of plasmid incompatibility groups IncHI2 (1 isolate), IncI2 (2 isolates), and combined IncHI2 and IncHI2A (2 isolates). Transposon IS*AplI*, initially described as carrying the *mcr-1* gene (*2*), was identified in 18 of 22 contigs.

**Figure:**
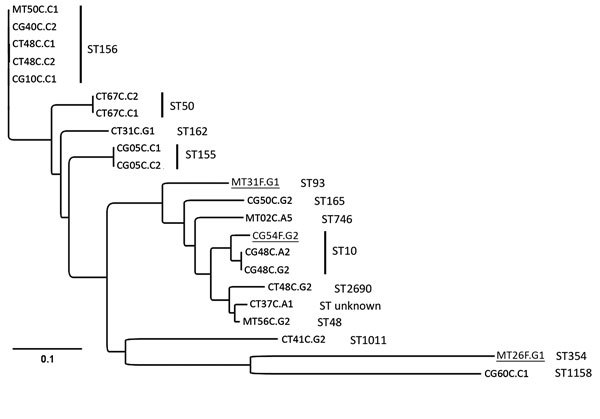
Phylogenetic analyses of *mcr-1*–positive *Escherichia coli* isolated from chickens and chicken farmers, Vietnam, 2012–2013. Maximum-likelihood tree of 22 *mcr-1*–carrying *E. coli* isolated from 15 chicken fecal samples and 3 human fecal swab samples (underlined), constructed by using CSI Phylogeny 1.4 (https://cge.cbs.dtu.dk//services/CSIPhylogeny/), shows a genome-wide single-nucleotide polymorphism (SNP) comparison. A total of 74,585 SNPs were concatenated for pairwise comparison (difference between pairs 0–32,267 SNPs). The multilocus sequence types (ST) are indicated next to the isolate names. The ST155 isolates CG05C.C1 and CG05C.C2 differ by 1 SNP; the ST10 isolates CG48C.A2 and CG48C.G2 differ by 1 SNP and 1 antimicrobial resistance gene; the ST156 isolates CT48C.C1 and CT48C.C2 differ by 4 SNPs and 3 antimicrobial resistance genes; and the ST50 isolates CT67C.C1 and CT67C.C2 are phenotypically different but have 0 SNP differences and originate from the same sample and are therefore likely to be highly related or identical. Scale bar indicates number of nucleotide substitutions per site.

We investigated risk factors for fecal colonization with *mcr-1*–carrying bacteria separately for small-scale farms and household farms because a joint model did not converge due to inflated sampling weight assigned to household chicken farms (Technical Appendix 1 Table 1). Multivariate analysis identified the presence of younger chickens (<20.5 weeks old) and the use of colistin as independent risk factors for fecal colonization with *mcr-1*–carrying bacteria in chickens (odds ratios [ORs] 21.3 and 5.1, respectively) in small-scale farms (Table 2). We were unable to identify potential risk factors associated with fecal colonization with *mcr-1*-carrying bacteria in chickens in household farms. Among human participants, farmers who were exposed to *mcr-1*–positive chickens showed a significantly increased risk for colonization with *mcr-1*–carrying bacteria (OR 5.3; Table 2) in contrast with urban individuals not involved in chicken farming, rural individuals not exposed to chickens, and farmers with *mcr-1*–negative chickens.

**Table 2:** Multivariate analysis of risk factors associated with fecal colonization with *mcr-1*–carrying bacteria in small-scale chicken farms (N = 94) and in humans (N = 440), Vietnam, 2012–2013*

Variables	No. tested	No. *mcr-1*–positive	OR (95% CI)	p value
Small-scale chicken farms				
Age of chickens				
Chickens <20.5 weeks old	47	32	21.3 (5.8–78.5)	<0.001
Chickens ≥20.5 weeks old	47	8	Referent	
Use of colistin	21	14	5.1 (1.4–18.8)	0.017
Humans				
Urban persons†	88	8	Referent	
Rural persons†	173	31	2.1 (0.9–5.0)	0.075
Farmers exposed to *mcr-1*–negative chickens	91	16	1.8 (0.7–4.7)	0.205
Farmers exposed to *mcr-1*–positive chickens	88	29	5.3 (2.2–12.7)	<0.001

*OR, odds ratio. †Not involved in poultry farming.

## Conclusions

Our study shows that colonization with *mcr-1*–carrying bacteria in chickens is associated with colistin usage and colonization of humans is associated with exposure to *mcr-1*–positive chickens. These findings suggest that colistin use is the main driver for the observed high prevalence (59.4%) of *mcr-1* in fecal samples from chickens, with zoonotic transmission explaining the high prevalence (34.7%) in farmers. Zoonotic transmission of colistin-resistant *E. coli* from a domesticated pig (*11*) and companion animals (*12*) to humans has been reported.

We found that younger chickens were more likely to be colonized with *mcr-1*–carrying bacteria than older chickens (>20.5 weeks), probably because of the higher antimicrobial treatment incidence in younger chickens (74.0 [interquartile range 0–278]/1,000 chickens treated daily with 1 defined daily dose) than in older chickens (46.3 [interquartile range 0–124]/1,000 chickens treated daily with 1 defined daily dose) (N.V. Trung, unpub. data). However, our study was insufficiently powered to detect such an association in multivariate analysis. In addition, the gastrointestinal tract of younger chickens might be colonized by antimicrobial-resistant bacteria more readily than older chickens (*13*).

The spread of the *mcr-1* gene on different plasmid types (IncI2, IncHI2, and IncHI2A) might explain its successful spread in different *E. coli* clones. We also identified the IS*Apl1* transposon in 81.8% (18/22) of our isolates. Because this genetic element is involved in horizontal gene transfer, it is likely to be a key factor contributing to the widespread dissemination of *mcr-1* (*14*).

Our study is subject to several limitations. First, the cross-sectional study design precludes the demonstration of direct transmission of the *mcr-1* gene between chickens and humans. Second, the presence of colistin in chicken feeds could not be verified and thus misclassification of farms in terms of their colistin use was possible. Last, we did not screen for the *mcr-2* gene, which is also involved in colistin resistance (*15*).

In summary, our results show an association between colistin use on farms and the presence of the *mcr-1* gene in animals. Given the potentially serious consequences of the spread of the *mcr-1* gene from food production animals to humans, prudent use of antimicrobial drugs in animal production should be enforced globally, including in small-scale and household farms.

Technical Appendix 1 Detailed materials and methods describing the selection and recruitment of study subjects, data collection, sample analyses, real-time PCR detection of the *mcr-1* gene, adjustment of prevalence estimates, whole-genome sequencing analyses, and risk factor analyses. Univariate analyses of the risk factors associated with the presence of *mcr-1*–carrying bacteria in chickens and humans in Vietnam in 2012−2013, comparison of characteristics of study participants and farms included and excluded from the risk factor analyses, and use of antimicrobial drugs in chickens and humans in southern Vietnam.

Technical Appendix 2 Characteristics of *mcr-1*–positive *Escherichia coli* from chickens and farmers, Vietnam, 2012–2013.
