# Supplementary material for: Zoonotic Transmission of mcr-1 Colistin Resistance Gene from Small-Scale Poultry Farms, Vietnam
Source: Emerg Infect Dis. 2017 Mar;23(3):529–32. doi: 10.3201/eid2303.161553 (PMC5382726; doi:10.3201/eid2303.161553)
Supplement: Technical Appendix 1 — Detailed materials and methods describing the selection and recruitment of study subjects, data collection, sample analyses, real-time PCR detection of the mcr-1 gene, adjustment of prevalence estimates, whole-genome sequencing analyses, and risk factor analyses. Univariate analyses of the risk factors associated with the presence of mcr-1–carrying bacteria in chickens and humans in Vietnam in 2012−2013, comparison of characteristics of study participants and farms included and excluded from the risk factor analyses, and use of antimicrobial drugs in chickens and humans in southern Vietnam. [file 16-1553-Techapp-s1.pdf]

Article DOI: http://dx.doi.org/10.3201/eid2303.161553

Zoonotic Transmission of *mcr-1* Colistin Resistance Gene from Small-Scale Poultry Farms, Vietnam

Technical Appendix

Selection and Recruitment of Study Subjects

Chicken Farmers and Farms

In our study, we included 102 household chicken farms (≥10–200 chickens) and 102 small-scale chicken farms (>200–2,000 chickens) stratified by district (n = 3; each having 34 household-scale and 34 small-scale farms). In each district, farms were selected randomly by using different approaches for small-scale and household-scale farms. Small-scale farms and farmers were randomly selected from the list of farms available at the Sub-Department of Animal Health in Tien Giang by using a random number table. To select household-scale farms and farmers within the chosen district, a commune was selected at random, and within the commune, the farm was selected as the first farm encountered 500 m from the center of the commune (direction was designated by spinning a bottle). Farmers and farms were included in the study if they fulfilled all the following inclusion criteria: 1) living in My Tho, Cho Gao, or Chau Thanh; 2) being healthy defined as not having been hospitalized in the past month, not currently having underlying serious chronic infectious diseases (e.g., TB), and having the ability to understand the informed consent form and questionnaire; 3) engaged in small-scale or household-scale chicken production; and 4) providing informed consent. Farmers and farms were excluded from the study if they did not fulfill any of the inclusion criteria.

Matched Persons Not Involved in Poultry Farming

We included 306 persons not involved in poultry farming in our study, which were matched by age and sex to the studied farmers (Technical Appendix Table 6). These consisted of 204 persons not involved in poultry farming from the same districts with the farmers (rural

persons) and 102 persons not involved in poultry farming from the provincial city (urban persons).

For every recruited farmer, we used multiple stages of sampling to identify matched persons with the ratio of 1:1 and 1:0.5 for rural persons and urban persons, respectively. In the first stage, we listed all persons that matched the age, sex, and location of the recruited farmer by viewing the registration population data available at the Preventive Medicine Department in Tien Giang. In the second stage, we randomly selected the person from the list of all persons by using a random number table. After the person was approached, we only included them in the study if they fulfilled all of the inclusion criteria. The inclusion criteria for rural persons were 1) providing informed consent; 2) living in Cho Gao, Chau Thanh, or the rural area of My Tho city; 3) living in a household where poultry is not raised and where poultry is not raised in the contiguous, immediately adjacent households; 4) not having worked on a farm raising poultry, in a slaughter line or slaughterhouse, or engaged in selling live or dead poultry over the past 12 months; 5) being healthy defined as not having been hospitalized in the past month, not currently having underlying serious chronic infectious diseases (e.g., TB), and having the ability to understand the informed consent form and questionnaire; and 6) matching a recruited farmer by age (±5 years), sex, and commune. The inclusion criteria for urban persons were 1) providing informed consent; 2) living in the urban area of the provincial city; 3) not having worked on a farm raising poultry, in a slaughter line or slaughterhouse, or engaged in selling live or dead poultry over the past 12 months; 4) being healthy defined as not having been hospitalized in the past month, not currently having underlying, serious chronic infectious diseases (e.g., TB), and having the ability to understand the informed consent form and questionnaire; and 5) matching a recruited farmer by age (±5 years) and sex. Those who did not fulfill all of the inclusion criteria were excluded from the study. Those who did not wish to participate were replaced by the next-best fit.

Data Collection

Data on human antimicrobial drug use during the month before the study visit, including the product's commercial name, packaging information, dosage, and duration of use, were collected for all participants as well as for all household members by medicine cabinet surveys

(Technical Appendix Table 8), which consisted of a structured questionnaire containing both open and closed questions. Data on antimicrobial use for chickens were similarly collected during interviews with the farmers by using a questionnaire as published previously (*1*). The medicine cabinet survey has been shown to be efficient for getting data on antimicrobial drugs use in the community (*2*). Use of an antimicrobial drug was defined as the reported use in the previous month or the presence of the antimicrobial drug in the medicine cabinet. All questionnaires on antimicrobial use were administered for chickens by the staff from the Sub-department of Animal Health and for humans by the staff from the Preventive Medicine Center.

Sample Analysis

Buffered peptone water (225 mL) was added to each chicken fecal sample in a different container and was manually shaken. A volume of 1 mL from each container was diluted 1:1000 in saline solution. Human rectal swabs were vortexed to release and suspend the sample in the liquid transportation medium and then 100 µL was diluted 1:100 in saline solution. Plating 50 µL of this dilution and incubating overnight at 37°C resulted in the growth of >100 separate colonies when plated onto MacConkey agar without antimicrobials or MacConkey agar supplemented with nalidixic acid (16 mg/L), ceftazidime (2 mg/L), or gentamicin (8 mg/L). A sweep from the full growth was collected and stored in glycerol at –20°C; afterwards, 5 randomly selected *Escherichia coli*–like colonies from the MacConkey agar without antimicrobials and 2 from each of 3 antimicrobial-supplemented agars were picked and subcultured for identification and antimicrobial susceptibility testing. Antimicrobial susceptibility testing was performed by using the disc diffusion method in accordance with the Clinical and Laboratory Standards Institute guidelines and breakpoints (*3*). Eleven antimicrobials were tested including tetracycline (30 mg), trimethoprim/sulfamethoxazole (1.25/23.75 mg), chloramphenicol (30 mg), gentamicin (10 mg), amikacin (30 mg), ciprofloxacin (5 mg), ampicillin (10 mg), amoxicillin/clavulanic acid (30 mg), ceftazidime (30 mg), ceftriaxone (30 mg), and meropenem (10 mg). Colistin was not included since interpretative breakpoints for disc susceptibility testing are not available with this antimicrobial. Quality controls for susceptibility testing and identification were performed every week according to the Clinical and Laboratory Standards Institute guidelines (*3*). Strains with an intermediate-susceptibility result were considered resistant. From each subject, all isolates with a unique, phenotypic, antimicrobial susceptibility patterns were stored for further analyses.

All *mcr-1*–positive *E. coli* isolates were tested for colistin susceptibility by using the ETEST (bioMérieux, Marcy l'Etoile, France) and interpreted in accordance with European Committee on Antimicrobial Susceptibility Testing breakpoints (*4*).

Real-Time PCR Detection of the *mcr-1* Gene

We designed a new forward primer (CLR5-qF1, 5′-TGACACTTATGGCACGGTCT-3′) by using primer3 V4 (*5*,*6*). When combined with the reverse primer described by Liu et al. (CLR5-R, 5′-CTTGGTCGGTCTGTAGGG-3) (*7*), it produced a 62-bp fragment, which allowed for faster detection by real-time PCR, when we used the LightCycler 480 SYBR Green I Master mix (Roche Diagnostics, Indianapolis, IN, USA) following the manufacturer's instructions and using 60°C as the annealing temperature on a Roche LightCycler 480 instrument (Roche Diagnostics, Indianapolis, IN, USA).

Whole-Genome Sequencing Analysis

Whole-genome sequencing was performed for all *mcr-1*–positive *E. coli* isolates. Bacterial DNA was extracted from fresh pure cultures by using either the Wizard Genomic DNA purification kit (Promega, Madison, WI, USA) or the Qiagen DNeasy Blood and Tissue kit (Qiagen, Hilden, Germany). Library preparation was done in accordance with the manufacturer's instructions (Illumina, San Diego, CA, USA) and sequenced by using Illumina MiSeq technology with 150 paired-end settings (Illumina, Inc., San Diego, CA, USA). The reads were checked by using fastqc (http://www.bioinformatics.babraham.ac.uk/projects/fastqc/) and trimmed using Trimmomatic V0.33 (*8*). De-novo genome assembly was performed with SPAdes 3.6 (*9*), and coverage was determined with Samtools v0.1.19 (*10*).

The presence of the *mcr-1* gene and the IS*Apl1* transposon in the genomes was determined by using Blastn (*11*) with the assembled scaffolds as database and the published sequences as query (*7*). Online services provided by the Center for Genomic Epidemiology (https://cge.cbs.dtu.dk/services/) were used for multilocus sequence typing according to the scheme developed by Achtman et al. (*12*), for identification of plasmid incompatibility group (*13*) and for detection of acquired antimicrobial resistance genes other than *mcr-1* (*14*). We analyzed clonal complexes using e-burst V3 (http://eburst.mlst.net/v3/enter_data/single/) (*15*)

and the Multilocus Sequence Types (MLST) database at the University of Warwick (http://mlst.warwick.ac.uk/mlst/dbs/Ecoli) (*16*). The phylogenetic relationship between the isolates was determined by whole-genome comparison. Single-nucleotide polymorphisms (SNPs) were called by aligning the sequence reads to the genome of the commensal, nonpathogenic *E. coli* SE15 (accession no. NC_013654.1) (*17*) and distance between the isolates was visualized by constructing a maximum-likelihood tree on the basis of the SNP alignments with the online tool CSI Phylogeny 1.2 with the recommended default settings (*18*). Raw read sequences and assembled contigs have been deposited in the European Nucleotide Archive (http://www.ebi.ac.uk/ena) under the accession numbers ERS1262218–ERS1262239 (study accession no. PRJEB14873).

Adjustment of Prevalence Estimates for Stratified Study Design

Since the study was designed as a stratified survey with a fixed number of farms and participants in each stratum, not all the study units (farms and participants in the 3 districts) had the same probability of being selected. The prevalence of fecal colonization with *mcr-1*–carrying bacteria in chickens and humans was adjusted for the stratified survey design by assigning a stratum-specific sampling weight (W_i) to each observation unit (farm or subject) and then by using the following equation: $W_i = N_T/N_i$, where N_T = the total number of chicken farms or humans in that study district and N_i = the number of farms or participants in each stratum sampled ($i = 1...7$) (Technical Appendix Tables 1 and 2). Standard errors were corrected to calculate the prevalence in each stratum. Sampling weight and sampling fraction of participants belonging to each study stratum were calculated under the assumption that chicken farmers accounted for 80% of the rural population.

Risk Factor Analysis

We built logistic regression models to investigate the risk factors associated with fecal colonization with *mcr-1*–carrying bacteria in chicken farms and human participants (Technical Appendix Tables 3–5). In the model for studying the risk factors in chickens, a total of 40 variables were first tested in univariate analyses, including factors describing the farms (production type and presence of other animals), farmers' demographic factors (Technical

Appendix Table 6), husbandry factors (Technical Appendix Table 7), and antimicrobial usage (Technical Appendix Table 8). We then excluded variables with <10 outcome events (*20*,*21*). Continuous variables such as the total number of chickens on the farm, the age of the chickens, the density of chickens on the farm, the age of the farmer, and the number of years experienced in chicken farming were stratified into 2 predefined categories by using the median of the obtained values as a cutoff value.

Similarly, for identifying risk factors associated with *mcr-1*–carrying bacteria in humans, a total of 9 variables were tested in univariate analyses, and participants were stratified into 2 categories on the basis of median age. Based on their biologic plausibility and a p value <0.15 in the univariate analyses, variables were considered for multivariable analysis and were included by using a stepwise forward approach (*22*). Variables were retained in the final models if the p value was <0.05. All biologically plausible variables were included in the final model. All interactions between final significant variables were tested. We performed all statistical analyses using R packages epicalc, survey, and adegenet (http://www.r-project.org).

References

1. Nguyen VT, Carrique-Mas JJ, Ngo TH, Ho HM, Ha TT, Campbell JI, et al. Prevalence and risk factors for carriage of antimicrobial-resistant *Escherichia coli* on household and small-scale chicken farms in the Mekong Delta of Vietnam. J Antimicrob Chemother. 2015;70:2144–52. PubMed

2. Hardon A, Hodgkin C, Fresle, D. How to investigate the use of medicines by consumers. 2004 [cited 2016 Sep 12]. http://www.who.int/medicines/areas/rational_use/Manual1_HowtoInvestigate.pdf

3. Clinical and Laboratory Standards Institute. Performance standards for antimicrobial susceptibility testing; twenty-first international supplement. CLSI document M100-S21. Wayne (PA): Clinical and Laboratory Standards Institute; 2011.

4. European Committee on Antimicrobial Susceptibility Testing. Breakpoint tables for interpretation of MICs and zone diameters. Version 6.0. 2016 JAN 01 [cited 2016 Sep 12]. http://www.eucast.org/fileadmin/src/media/PDFs/EUCAST_files/Breakpoint_tables/v_6.0_Breakpoint_table.pdf

5. Koressaar T, Remm M. Enhancements and modifications of primer design program Primer3. Bioinformatics. 2007;23:1289–91. PubMed http://dx.doi.org/10.1093/bioinformatics/btm091

6. Untergasser A, Cutcutache I, Koressaar T, Ye J, Faircloth BC, Remm M, et al. Primer3—new capabilities and interfaces. Nucleic Acids Res. 2012;40:e115. PubMed http://dx.doi.org/10.1093/nar/gks596

7. Liu YY, Wang Y, Walsh TR, Yi LX, Zhang R, Spencer J, et al. Emergence of plasmid-mediated colistin resistance mechanism MCR-1 in animals and human beings in China: a microbiological and molecular biological study. Lancet Infect Dis. 2016;16:161–8. PubMed

8. Bolger AM, Lohse M, Usadel B. Trimmomatic: a flexible trimmer for Illumina sequence data. Bioinformatics. 2014;30:2114–20. PubMed http://dx.doi.org/10.1093/bioinformatics/btu170

9. Bankevich A, Nurk S, Antipov D, Gurevich AA, Dvorkin M, Kulikov AS, et al. SPAdes: a new genome assembly algorithm and its applications to single-cell sequencing. J Comput Biol. 2012;19:455–77. PubMed http://dx.doi.org/10.1089/cmb.2012.0021

10. Li H, Handsaker B, Wysoker A, Fennell T, Ruan J, Homer N, et al.; 1000 Genome Project Data Processing Subgroup. The sequence alignment/map format and SAMtools. Bioinformatics. 2009;25:2078–9. PubMed http://dx.doi.org/10.1093/bioinformatics/btp352

11. Altschul SF, Gish W, Miller W, Myers EW, Lipman DJ. Basic local alignment search tool. J Mol Biol. 1990;215:403–10. PubMed http://dx.doi.org/10.1016/S0022-2836(05)80360-2

12. Larsen MV, Cosentino S, Rasmussen S, Friis C, Hasman H, Marvig RL, et al. Multilocus sequence typing of total-genome-sequenced bacteria. J Clin Microbiol. 2012;50:1355–61. PubMed http://dx.doi.org/10.1128/JCM.06094-11

13. Carattoli A, Zankari E, García-Fernández A, Voldby Larsen M, Lund O, Villa L, et al. In silico detection and typing of plasmids using PlasmidFinder and plasmid multilocus sequence typing. Antimicrob Agents Chemother. 2014;58:3895–903. PubMed http://dx.doi.org/10.1128/AAC.02412-14

14. Zankari E, Hasman H, Cosentino S, Vestergaard M, Rasmussen S, Lund O, et al. Identification of acquired antimicrobial resistance genes. J Antimicrob Chemother. 2012;67:2640–4. PubMed http://dx.doi.org/10.1093/jac/dks261

15. Feil EJ, Li BC, Aanensen DM, Hanage WP, Spratt BG. eBURST: inferring patterns of evolutionary descent among clusters of related bacterial genotypes from multilocus sequence typing data. J Bacteriol. 2004;186:1518–30. PubMed http://dx.doi.org/10.1128/JB.186.5.1518-1530.2004

16. Wirth T, Falush D, Lan R, Colles F, Mensa P, Wieler LH, et al. Sex and virulence in *Escherichia coli*: an evolutionary perspective. Mol Microbiol. 2006;60:1136–51. PubMed http://dx.doi.org/10.1111/j.1365-2958.2006.05172.x

17. Toh H, Oshima K, Toyoda A, Ogura Y, Ooka T, Sasamoto H, et al. Complete genome sequence of the wild-type commensal *Escherichia coli* strain SE15, belonging to phylogenetic group B2. J Bacteriol. 2010;192:1165–6. PubMed http://dx.doi.org/10.1128/JB.01543-09

18. Kaas RS, Leekitcharoenphon P, Aarestrup FM, Lund O. Solving the problem of comparing whole bacterial genomes across different sequencing platforms. PLoS One. 2014;9:e104984. PubMed http://dx.doi.org/10.1371/journal.pone.0104984

19. Statistical Office of Tien Giang Province. Statistical yearbook Tien Giang province 2011. Ho Chi Minh City (Vietnam): Statistical Printing Factory; 2012.

20. Peduzzi P, Concato J, Feinstein AR, Holford TR. Importance of events per independent variable in proportional hazards regression analysis. II. Accuracy and precision of regression estimates. J Clin Epidemiol. 1995;48:1503–10. PubMed http://dx.doi.org/10.1016/0895-4356(95)00048-8

21. Peduzzi P, Concato J, Kemper E, Holford TR, Feinstein AR. A simulation study of the number of events per variable in logistic regression analysis. J Clin Epidemiol. 1996;49:1373–9. PubMed http://dx.doi.org/10.1016/S0895-4356(96)00236-3

22. Hosmer DW, Lemeshow S, Sturdivant RX. Applied logistic regression. 3rd ed. Hoboken (NJ): John Wiley & Sons; 2004.

Technical Appendix Table 1. Sampling weight and sampling fraction of chicken farms, Tien Giang province, Vietnam, 2012–2013*

Stratum	NT†	Ni	Fraction that should be sampled	Fraction sampled	Wi
Chau Thanh household farm	10,762	34	0.3697	0.00117	317
Cho Gao household farm	16,101	34	0.5532	0.00117	474
My Tho household farm	2,026	34	0.0696	0.00117	60
Chau Thanh small farm	36	34	0.0012	0.00117	1
Cho Gao small farm	147	34	0.0051	0.00117	4
My Tho small farm	34	34	0.0012	0.00117	1

*Ni, no. of farms sampled per stratum; NT, no. of farms per stratum; Wi, sampling weight.
†Tien Giang statistical office (*19*).

Technical Appendix Table 2. Sampling weight and sampling fraction of participants, Tien Giang province, Vietnam, 2012–2013*

Stratum	NT†	Ni	Fraction that should be sampled	Fraction sampled	Wi
My Tho, rural	16,621	68	0.027	0.000111	244
My Tho, farmer	66,486	68	0.108	0.000111	978
Chau Thanh, rural	46,067	68	0.075	0.000111	677
Chau Thanh, farmer	184,266	68	0.301	0.000111	2,710
Cho Gao, rural	33,594	68	0.055	0.000111	494
Cho Gao, farmer	134,375	68	0.219	0.000111	1,976
My Tho, urban	131,650	102	0.215	0.000166	1,291

*Ni, no. of farms sampled per stratum; NT, no. of farms per stratum; Wi, sampling weight.
†Tien Giang statistical office (*19*).

Technical Appendix Table 3. Univariate analyses of risk factors associated with *mcr-1*–carrying bacteria in small-scale chicken farms (N = 94), Vietnam, 2012–2013*

Risk factor	*mcr-1*–positive farm	Total	%	Odds ratio	95% CI	p value
Male farmer	28	51	54.9	1.94	0.61–6.14	0.261
Age of farmer						
<44 years	22	45	48.9	1.67	0.61–4.56	0.322
≥44 years	18	49	36.7	Ref	Ref	Ref
Experience in chicken farming						
<5 years	19	37	51.4	Ref	Ref	Ref
≥5 years	21	57	36.8	0.64	0.23–1.77	0.391
Level of education attained by the farmer						
Secondary school or less	6	17	35.3	Ref	Ref	Ref
Higher than secondary school	34	77	44.2	1.03	0.29–3.7	0.968
Location of farm						
My Tho city	11	32	34.4	Ref	Ref	Ref
Cho Gao district	17	32	53.1	2.16	0.79–5.96	0.138
Chau Thanh district	12	30	40.0	1.27	0.45–3.59	0.649
Type of chicken production						
Eggs	13	56	23.2	Ref	Ref	Ref
Meat	27	38	71.1	10.36	3.29–32.69	<0.0001
Total number of chickens						
200–1400	19	45	42.2	Ref	Ref	Ref
1400–2000	21	49	42.9	1.13	0.42–3.05	0.814
Chicken density						
<10 chickens/m^2	21	49	42.9	Ref	Ref	Ref
≥10 chickens/m^2	19	45	42.2	1.17	0.44–3.15	0.752
Age of chickens						
<20.5 weeks	32	47	68.1	19.39	5.8–4.8	<0.0001
≥20.5 weeks	8	47	17.0	Ref	Ref	Ref
Chickens confined in pen/house 24 h/day	35	84	41.7	0.64	0.13–3.22	0.589
All-in-all-out system	25	60	41.7	1.21	0.43–3.44	0.716
Farms that bought day-old chickens	38	81	46.9	4.35	0.68–28.04	0.125
Source of day-old chickens						
Hatched in farm	5	11	45.5	Ref	Ref	Ref
Local hatchery	11	16	68.8	6.27	1.02–38.59	0.051
Company hatchery	23	55	41.8	3.34	0.79–14.09	0.104
Presence of other animals on farms						
Any	39	88	44.3	2.72	0.27–27.01	0.395
Fighting cocks	5	10	50.0	2.07	0.43–10.01	0.369
Ducks	8	24	33.3	0.58	0.18–1.92	0.376
Pigs	20	41	48.8	1.45	0.53–3.97	0.472
Cattles	7	15	46.7	0.9	0.26–3.08	0.861
Dogs	34	74	45.9	4.35	1.18–16.09	0.03
Cats	20	46	43.5	0.93	0.34–2.51	0.882
Fish pond	21	48	43.8	1.17	0.43–3.18	0.753
Farms that used antimicrobials	31	68	45.6	0.97	0.33–2.89	0.958
How often the farmer read guideline of antimicrobials						
Always	36	83	43.4	Ref	Ref	Ref
Sometimes	4	8	50.0	2.75	0.55–13.65	0.22
Never	0	3	0	0	0–0	<0.001
Farms that used disinfectants	40	94	42.6	0.92	0.56–1.51	0.741
Wild bird seen at farm						
Never	11	20	55.0	Ref	Ref	Ref
Sometimes	27	68	39.7	0.45	0.14–1.5	0.198
Always	2	6	33.3	0.11	0.01–0.91	0.0429
Rodent seen at farm						
Never	11	21	52.4	Ref	Ref	Ref
Sometimes	25	61	41.0	0.57	0.17–1.92	0.365
Always	4	12	33.3	0.14	0.03–0.77	0.025
Farms that used commercial feed	40	93	43.0	1,600,119	211,647.3–12,097,395	<0.001
Farms with the presence of ante-room	4	4	100.0	19,058,486.62	5,121,705.8–70,918,933.3	<0.001
Change boot/shoes before entering pen/house	38	84	45.2	1.12	0.26–4.78	0.881
Foot bath/foot dip at entrance	29	73	39.7	0.7	0.21–2.33	0.565
Outsiders allowed in farm	0	2	0	0	0–0	<0.001
Use of specific antimicrobials at farm						

Risk factor	*mcr-1*–positive farm	Total	%	Odds ratio	95% CI	p value
Male farmer	28	51	54.9	1.94	0.61–6.14	0.261
Aminoglycosides	3	5	60.0	1.33	0.15–11.99	0.802
Penicillins	11	18	61.1	2.39	0.74–7.71	0.148
Lincosamides	1	1	100.0	1,895,525.65	250,674.27–14,333,411.8	<0.001
Macrolides	13	29	44.8	0.53	0.18–1.57	0.257
Colistin	14	21	66.7	3.7	1.18–11.58	0.026
Phenicols	3	11	27.3	0.88	0.18–4.4	0.875
Quinolones	5	9	55.6	2.39	0.45–12.64	0.308
Sulfonamides	4	6	66.7	4.04	0.59–27.76	0.159
Tetracyclines	11	31	35.5	0.32	0.11–0.97	0.047

*Ref, referent.

Technical Appendix Table 4. Univariate analyses of risk factors associated with *mcr-1*–carrying bacteria in household chicken farms (N = 94), Vietnam, 2012–2013*

Risk factor	*mcr-1*–positive farm	Total	%	Odds ratio	95% CI	p value
Male farmer	24	40	60.0	1.79	0.65–4.98	0.264
Age of farmer						
<46.5 years	23	47	48.9	Ref	Ref	Ref
≥46.5 years	30	47	63.8	1.49	0.56–3.98	0.428
Experience in chicken farming						
<8 years	25	45	55.6	Ref	Ref	Ref
≥8 years	25	49	51.0	0.57	0.21–1.49	0.252
Level of education attained by the farmer						
Secondary school or less	19	32	59.4	Ref	Ref	Ref
Higher than secondary school	34	62	54.8	1.16	0.42–3.22	0.774
Location of farm						
My Tho city	20	32	62.5	Ref	Ref	Ref
Cho Gao district	17	30	56.7	1.67	0.61–4.54	0.32
Chau Thanh district	16	32	50.0	1.31	0.48–3.58	0.603
Type of chicken production						
Eggs	0	1	0	Ref	Ref	Ref
Meat	53	93	57.0	11,225,671	1,489,675–84,592,729	<0.001
Total no. of chickens						
<75	27	47	57.4	1.14	0.42–3.09	0.792
75–199	26	47	55.3	Ref	Ref	Ref
Chicken density						
<1 chickens/m^2	31	57	54.4	Ref	Ref	Ref
≥1 chickens/m^2	22	37	59.5	1.42	0.54–3.75	0.478
Age of chickens						
<16 weeks	23	45	51.1	Ref	Ref	Ref
≥16 weeks	30	49	61.2	1.41	0.53–3.73	0.487
Chickens confined in pen/house 24h/day	1	1	100.0	1,052,607.31	139,818.2–7,924,448.9	<0.001
All-in-all-out system	20	29	69.0	1.98	0.71–5.57	0.197
Farms that bought day-old chickens	51	92	55.4	0	0–0	<0.001
Source of day-old chickens						
Hatched in farm	26	54	48.1	Ref	Ref	Ref
Local hatchery	14	21	66.7	2.87	0.86–9.6	0.09
Company hatchery	6	8	75.0	2.27	0.38–13.59	0.373
Market/neighbor	6	10	60.0	1.35	0.29–6.4	0.704
Presence of other animals on farm						
Any	53	94	56.4	NC	NC–NC	NC
Fighting cocks	17	28	60.7	0.95	0.32–2.78	0.926
Ducks	28	42	66.7	2.44	0.89–6.72	0.087
Pigs	30	48	62.5	1.86	0.7–4.92	0.217
Cattles	13	21	61.9	1.47	0.43–4.99	0.539
Dogs	51	89	57.3	2.46	0.26–23.61	0.438
Cats	30	53	56.6	0.62	0.23–1.68	0.352
Fish pond	32	59	54.2	0.73	0.28–1.94	0.531
Using antimicrobials on farm	26	44	59.1	1.27	0.48–3.34	0.627
How often the farmer read guideline of antimicrobials						
Always	41	71	57.7	Ref	Ref	Ref

Risk factor	*mcr-1*–positive farm	Total	%	Odds ratio	95% CI	p value
Male farmer	24	40	60.0	1.79	0.65–4.98	0.264
Sometimes	10	19	52.6	0.84	0.25–2.89	0.788
Never	2	4	50.0	0.77	0.08–7.83	0.828
Farms that used disinfectants	48	85	56.5	0.89	0.15–5.4	0.902
Wild bird seen in farm						
Never	22	38	57.9	Ref	Ref	Ref
Sometimes	29	50	58.0	0.86	0.31–2.37	0.77
Always	2	6	33.3	0.35	0.04–3.44	0.369
Rodent seen in farm						
Never	23	41	56.1	Ref	Ref	Ref
Sometimes	28	47	59.6	0.71	0.26–1.92	0.503
Always	2	6	33.3	1.93	0.3–12.6	0.493
Farms that used commercial feed	39	64	60.9	1.69	0.56–5.09	0.354
Farms with the presence of ante-room	2	2	100.0	14,362,266.53	3,328,276.24–61,976,436.25	<0.001
Change boot/shoes before entering pen/house	31	50	62.0	2.21	0.84–5.81	0.113
Foot bath/foot dip at entrance	25	40	62.5	1.89	0.71–5.07	0.207
Outsiders allowed in farm	7	13	53.8	0.73	0.17–3.12	0.67
Use of specific antimicrobials on farm						
Aminoglycosides	5	9	55.6	0.87	0.15–4.99	0.874
Penicillins	5	12	41.7	0.93	0.24–3.65	0.915
Lincosamides	4	4	100.0	13,773,734.42	4,583,095.04–41,394,681.58	<0.001
Macrolides	9	10	90.0	41.17	4.74–357.48	0.001
Colistin	9	18	50.0	0.66	0.2–2.23	0.51
Phenicols	0	2	0	0	0–0	<0.001
Quinolones	5	8	62.5	0.99	0.19–5.09	0.99
Sulfonamides	1	5	20.0	0.12	0.01–1.15	0.068
Tetracyclines	14	21	66.7	1.67	0.51–5.43	0.397

*NC, not calculated; ref, referent.

Technical Appendix Table 5. Univariate analyses of risk factors associated with *mcr-1*–carrying bacteria in humans (N = 440), Vietnam, 2012–2013*

Risk factors	*mcr-1*–positive subject	Total	%	Odds ratio	95% CI	p value
Participant group						
Farmers exposed to *mcr-1*–negative chickens	16	91	17.6	1.84	0.72–4.69	0.205
Farmers exposed to *mcr-1*–positive chickens	29	88	33.0	5.31	2.23–12.65	<0.001
Rural person not exposed to chickens	31	173	17.9	2.14	0.93–4.96	0.07
Urban person not exposed to chickens	8	88	9.1	Ref	Ref	Ref
Household location						
Cho Gao district	25	143	17.5	1	0.51–1.96	1
Chau Thanh district	30	147	20.4	1.37	0.72–2.61	0.34
My Tho city	29	150	19.3	Ref	Ref	Ref
Age of participant						
<46 years	45	214	21.0	1.56	0.88–2.76	0.13
≥46 years	39	226	17.3	Ref	Ref	Ref
Male participant	55	283	19.4	1.06	0.59–1.92	0.84
Presence of other animals	62	298	20.8	2.16	1.17–3.99	0.01
Presence of pig(s)	27	93	29.0	2.12	1.16–3.86	0.01
Participants that used antimicrobials in the past month	15	69	21.7	1.02	0.49–2.11	0.96
Chicken meat consumption						
Often (at least twice/week)	35	198	17.7	1.68	0.34–8.17	0.52
Sometimes (at least twice/month)	45	209	21.5	1.67	0.37–7.44	0.5
Never	4	33	12.1	Ref	Ref	Ref
Egg consumption						
Often (at least twice/week)	17	82	20.7	2.47	0.61–9.94	0.2
Sometimes (at least twice/month)	63	334	18.9	3.02	0.75–12.11	0.11
Never	4	24	16.7	Ref	Ref	Ref

*Ref, referent.

Technical Appendix Table 6. Comparison of key characteristics of study participants in Tien Giang province, Vietnam in 2012–2013

	Included human participants			Excluded human participants†		
Participant characteristic	Farmers, N = 179	Rural persons, N = 173	Urban persons, N = 88	Farmers, N = 25	Rural persons, N = 31	Urban persons, N = 14
Median age, y (IQR)	45.0 (38.5–54.0)	46.0 (38.0–53.0)	46.5 (42.0–53.0)	42.0 (36.0–52.0)	48.0 (37.5–55.0)	46.0 (40.8–58.5)
Male participant, no. (%)	116 (64.8)	116 (67.1)	51 (58.0)	16 (64.0)	18 (58.1)	9 (64.3)
Location of household						
Cho Gao district, no. (%)	57 (31.8)	57 (32.9)	29 (33.0)	11 (44.0)	11 (35.5)	5 (35.7)
Chau Thanh district, no. (%)	58 (32.4)	58 (33.5)	31 (35.2)	10 (40.0)	10 (32.3)	3 (21.4)
My Tho city, no. (%)	64 (35.8)	58 (33.5)	28 (31.8)	4 (16.0)	10 (32.3)	6 (42.9)

*IQR, interquartile range.
†Participants were excluded from risk factor analysis if sweep samples had not been stored (n = 45) or did not show any growth on MacConkey agar plate (n = 25) and therefore could not be tested for the presence of the *mcr-1* gene.

Technical Appendix Table 7. Comparison of key characteristics of study farms, Tien Giang province, Vietnam, 2012–2013

Characteristic	Included farms, N = 188	Excluded farms,* N = 16
Backyard-scale farm (%)	94 (50.0)	8 (50.0)
Type of chickens		
Meat, no. (%)	108 (57.4)	8 (50.0)
Egg, no. (%)	57 (30.3)	6 (37.5)
Mixed, no. (%)	23 (12.2)	2 (12.5)
Location of farm		
Cho Gao district, no. (%)	64 (34.0)	4 (25.0)
Chau Thanh district, no. (%)	60 (31.9)	8 (50.0)
My Tho city, no. (%)	64 (34.0)	4 (25.0)

* Farms were excluded from risk factor analysis if sweep samples had not been stored and therefore could not be tested for the presence of the *mcr-1* gene.

Technical Appendix Table 8. Use of antimicrobial drugs in chickens and humans, Tien Giang province, Vietnam, 2012–2013

		Humans,† no. (%)		
Class of antimicrobial	Chickens,* no. (%), N = 204	Farmer, N = 204	Rural, N = 204	Urban, N = 102
Any antimicrobial drug	118 (57.8)	33 (16.2)	32 (15.7)	17 (16.7)
1st generation cephalosporin	0 (0)	12 (5.9)	17 (8.3)	7 (6.9)
2nd generation cephalosporin	0 (0)	1 (0.5)	2 (1.0)	1 (1.0)
3rd generation cephalosporin	0 (0)	5 (2.5)	6 (2.9)	1 (1.0)
Penicillins	32 (15.7)	11 (5.4)	6 (2.9)	5 (4.9)
Polymyxins	39 (19.1)	0 (0)	0 (0)	0 (0)
Macrolides	38 (18.6)	3 (1.5)	4 (2)	4 (3.9)
Quinolones	19 (9.3)	2 (1.0)	2 (1.0)	1 (1.0)
Lincosamides	4 (2.0)	1 (0.5)	0 (0)	1 (1.0)
Aminoglycosides	18 (8.8)	1 (0.5)	0 (0)	0 (0)
Chloramphenicol	0 (0)	1 (0.5)	0 (0)	0 (0)
Phenicols	12 (5.9)	0 (0)	0 (0)	0 (0)
Sulfonamides/trimethoprim	12 (5.9)	0 (0)	0 (0)	1 (1.0)
Tetracyclines	51 (25.0)	0 (0)	0 (0)	1 (1.0)
Pleuromutilins	1 (0.5)	0 (0)	0 (0)	0 (0)

*Use during the previous 3 months for household-scale farms (≥10–200 chickens) or for the current flock for small-scale farms (>200–2000 chickens).
†Use during the month before the survey visit.
